# Understanding Nephrogenic Systemic Fibrosis

**DOI:** 10.1155/2012/912189

**Published:** 2012-11-04

**Authors:** Tushar Chopra, Kiran Kandukurti, Silvi Shah, Raheel Ahmed, Mandip Panesar

**Affiliations:** Division of Nephrology, Department of Internal Medicine, State University of NY at Buffalo, Buffalo, NY 14215, USA

## Abstract

Nephrogenic systemic fibrosis (NSF) is a rare and a debilitating disease noted uncommonly in patients with impaired renal function when exposed to low-stability gadolinium-based contrast agents (Gd-CAs). According to experimental studies, cytokines released by the stimulation of effector cells such as skin macrophages and peripheral blood monocytes activate circulating fibroblasts which play a major role in the development of NSF lesions. The presence of permissive factors, presumably, provides an environment conducive to facilitate the process of fibrosis. Multiple treatment modalities have been tried with variable success rates. More research is necessary to elucidate the underlying pathophysiological mechanisms which could potentially target the initial steps of fibrosis in these patients. This paper attempts to collate the inferences from the in vivo and in vitro experiments to the clinical observations to understand the pathogenesis of NSF. Schematic representations of receptor-mediated molecular pathways of activation of macrophages and fibroblasts by gadolinium and the final pathway to fibrosis are incorporated in the discussion.

## 1. Introduction

Nephrogenic systemic fibrosis (NSF) is a fibrosing disorder which predominantly occurs in patients whose estimated glomerular filtration rate is below 30 mL/min/1.73 m^2^ or who are on dialysis [1, 2]. Clinically, these patients present with a thickening and hardening of skin which is often associated with pain, muscle weakness, bone pain, and joint contractures leading to severe disability [3]. These lesions can be found all over the body but typically occur on the lower extremities and the face is usually spared [4]. NSF, first described in 1997, can affect both children and the elderly and has an average age of onset of 46.8 years [5, 6]. No genetic or racial predisposition has been established. 

The most common presenting morphology is sharply demarcated plaques (58%) with irregular edges but papules (32%), nodules (17%), and rarely macules, vesicles, blisters, bullae, and ulcers have also been reported [1, 4, 5, 7]. The lesions are progressive and later can evolve into wrinkles and woody skin with a cobblestone or “peau d'orange” texture [8]. NSF skin lesions are usually hyperpigmented and erythematous (39%) but can vary from violaceous, brown, tan, yellow, pink, orange-red, and grey-brown [4, 9]. Although initially named, “Nephrogenic Fibrosing Dermopathy,” since it was thought that the lesions were limited to the skin, it is now well documented that these lesions extend beyond the dermis and can involve joints, skeletal muscles, testes, kidney, myocardium, and dura [10–12]. A diagnosis can be made by skin or muscle biopsy and histopathology. Recently, a clinicopathological system to aid in the diagnosis of NSF has been proposed [13]. Clinically, NSF may resemble scleromyxedema but there are distinct histological features that set it apart. NSF lesions include proliferation of spindle shaped dermal cells, which may extend into subcutaneous tissue, thickened collagen bundles with surrounding clefts, increased elastic fibers and increased dermal mucin deposition. The spindle cells may demonstrate long dendritic processes and most of these cells are immunohistochemically positive for CD34 and procollagen I [1, 14]. They may also stain for CD68 implying a histiocyte differentiation. Some stellate cells are positive for factor XIIIa and represent an overabundance of resident cells of the papillary and periadnexal dermis. Inflammatory cells are usually absent but sometimes small clusters of perivascular mononuclear cells may be seen [15]. The histological features may evolve depending on the age of the lesion. The earliest findings consist of diffuse increase in fibroblasts, slight increase in dermal mucin and elastic fibers, and narrow collagen bundles with prominent interlaying clefts and associated edema. Late-disease lesions appear with thickening of collagen fibers and less clefting, more dermal mucin, more elastic fibres and fibroblasts, loose aggregates of CD68 positive histiocytes, and factor XIIIa positive dendrocytes [1]. 

There is no gold standard for the treatment of NSF. Cowper and Knopp reported in 2008 that the most effective therapy was maximization of renal function [16]. Oral or topical steroids [17, 18], high-dose intravenous immunoglobulins [19], calcipotriene ointment [20], cyclophosphamide [21], cyclosporine [22], thalidomide, interferon-alpha, psoralen, pentoxifylline [23], methotrexate [24], plasmapharesis [25], photodynamic therapy, ultraviolet light [26], and etanercept [27] have all been tried with variable success. Imatinib mesylate and extracorporeal photopheresis have also been used for the treatment of NSF [28, 29]. More recently, Alefacept has shown improvement in the cutaneous disease of three patients with NSF [30]. There is growing interest in renal transplantation and immunosuppressive medications as possible treatment modalities for NSF. Clinical improvement has been reported with renal transplantation [31] but the experience has not been universal [32, 33]. All the above treatment modalities have shown success only in a limited number of patients. 

## 2. Gadolinium-Based Contrast Agents (Gd-CAs)

Gadolinium-based contrast agents (Gd-CAs) are chelates containing gadolinium (Gd^3+^) ion. The chelates in the Gd-CAs complex reduce the toxicity associated with free Gd^3+^ ion. The use of Gd-CAs in advanced chronic kidney disease (CKD) is associated with the development of nephrogenic systemic fibrosis. A meta-analysis of six retrospective studies revealed an odds ratio of 26.7 (95% CI 10.27–69.24) of developing NSF when exposed to Gd-CAs in advanced CKD [34]. Gd-CAs are classified structurally as linear or cyclic, and chemically as ionic or nonionic. The non-ionic linear Gd-CAs are associated with higher risk of NSF due to lower thermodynamic and conditional stability constant values which renders them less stable (and hence are more likely to dechelate) than macrocyclic Gd-CAs [35]. A retrospective study of 190 reported cases of NSF demonstrated that 82% of the lesions were caused by a non-ionic linear Gd-CAs called gadodiamide (Omniscan, GE Healthcare) [36]. An overwhelming majority of cases have been reported following the use of Omniscan. The commonly used dose of Gd-CAs in magnetic resonance imaging contrast procedures is 0.1 to 0.3 mmol/kg. Subtotal nephrectomized (SNx) rats repeatedly exposed to high-dose (2.5 mmol/kg) nonformulated gadodiamide (i.e., without the 25 mM excess ligand caldiamide) developed worse skin lesions compared to gadoterate (Dotarem, Guerbet, Villepinte, France), a cyclic Gd-CA. Importantly, the high dose of 2.5 mmol/kg used in rats is equivalent to 0.4 mmol/kg in humans after adjusting for differences in body surface area and excretion half life (*t *_1/2_) of Gd-CAs between the two species. The excretion *t *_1/2_ of Gd-CAs is approximately 20 minutes in rats compared to 1.2–2 hours in humans [37]. Fretellier et al. also quantified the dissociated Gd^3+^ chelated Gd, and demonstrated in vivo transmetallation (dechelation) in SNx rats. The authors compared Omniscan (GE Healthcare, Chalfont St Giles, UK), nonformulated gadodiamide (i.e., without excess ligand, caldiamide), gadoterate (Dotarem, Guerbet, Villepente, France), and saline. They showed that the stability (determined as total concentration of Gd) of the cyclic Gd-CA, gadoterate, was lower in the skin and femur of gadoterate-treated rats when compared to rats treated with linear chelates. They also studied in vivo dechelation by the relaxometry method, which revealed gradual release of dissociated Gd^3+^ in soluble form in Omniscan and nonformulated gadodiamide-treated rats [38]. The United States Food and Drug Administration (US FDA) issued a warning in 2010, contraindicating the use of high-risk Gd-CAs, that is, Magnevist (Bayer Healthcare Pharmaceuticals, Inc), Omniscan (GE Healthcare, Inc) and Optimark (Mallinckrodt, Inc) in patients with acute kidney injury (AKI) and chronic, severe kidney disease. The European Medicines Agency (EMEA) had also warned against the use of the above contrast agents in 2009 in patients with severe kidney problems, neonates and in patients who are scheduled for or have recently received a liver transplant.

## 3. Pathophysiology of NSF 

**Figure 1:**
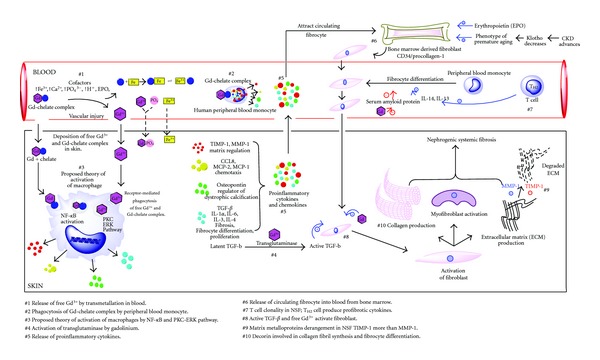
Schematic representation of the proposed pathophysiology of NSF.

A large number of in vitro and in vivo studies have been performed in order to explain the pathophysiology of NSF. In the following section, we have attempted to amalgamate the various theories that have been proposed so far to explain this complex mechanism. It is presumed that low stability Gd-chelate complex in the presence of permissive factors could either dissociate to release free Gd ions by the process of transmetallation or could remain intact in the complex form. Cytokines are released either by the free Gd ions stimulating skin macrophages or Gd-chelate complex stimulating the peripheral blood monocytes (PBMC) (Figure 1). This process leads to the stimulation of fibroblasts, eventually resulting in the characteristic fibrotic lesions which clinically present as thickening of skin with associated joint contractures. 

## 4. Macrophage Activation 

**Figure 2:**
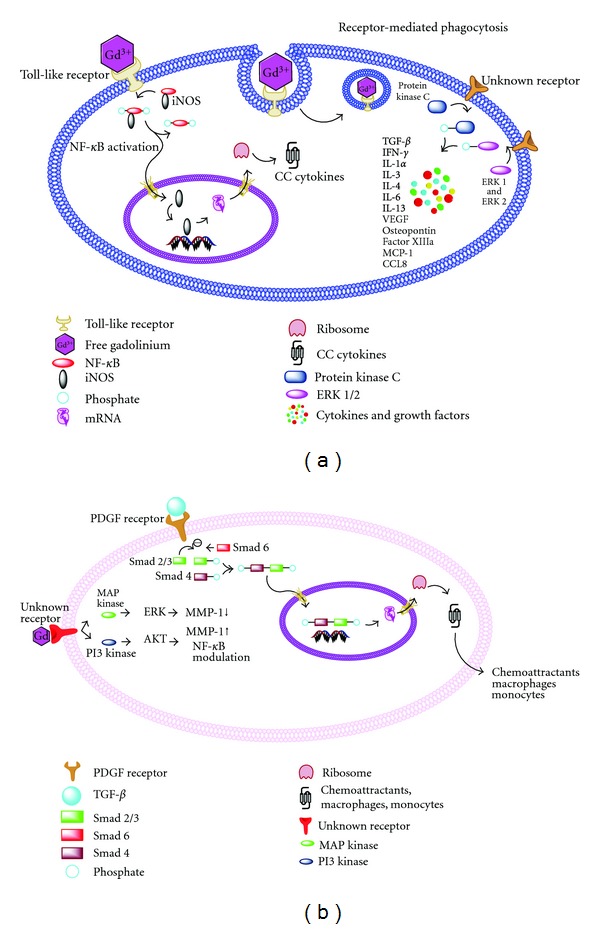
Schematic representation of activation of a macrophage and a fibroblast.

An activated macrophage is an important cell studied in NSF. Perazella et al. proposed that free gadolinium can leak into the tissues in the presence of endothelial dysfunction and vascular injury [39]. In vitro kinetic studies have demonstrated that free gadolinium facilitates precipitation of calcium and phosphate. This precipitation promotes macrophages to engulf both free Gd^3+^ as well as Gd-chelate complex by receptor-mediated phagocytosis. The intracellular pathways that have been linked to the activation of a macrophage in in vitro studies are the mitogen-activated protein kinase/extracellular signal-regulated kinase (MAPK/ERK) pathway and the nuclear factor-kappa B (NF- *κ* B) pathway. Gou et al. studied fetal bovine serum cells incubated with various concentrations of gadolinium chloride and demonstrated a 2.4-fold increase in transforming growth factor-beta (TGF-*β*) and an 18% increase in interleukin-6 (IL-6) production. The levels of these cytokines became significantly (*P* < 0.05) attenuated with the presence of protein kinase C (PKC), ERK1 and ERK2 inhibitors. Western blot analysis also revealed elevated levels of protein expression of PKC, ERK1 and ERK2 in the fetal bovine serum cells when treated with gadolinium chloride [40]. Del Galdo et al. showed upregulation of NF- *κ* B nuclear expression, chemokine ligand-8/matrix chemoattractant protein 1 (CCL8/MCP1) release and attenuation of inducible nitric oxide synthase (iNOS) protein levels in the presence of an NF-*κ*B peptide inhibitor following in vitro macrophage exposure to high-dose (50 m M) Omniscan (Figure 2(a)) [41]. Del Galdo et al. also showed induction of chemokine synthesis in macrophages by the activation of the toll-like receptor (TLR) pathway, in response to very high concentrations (50 mM) of Omniscan (Figure 2(a)) [42]. Ribonucleic acid (RNA) analysis and quantification of protein expression from four samples of terminally differentiated human macrophages exposed to either high-dose Omniscan or saline for 24 hours demonstrated an upregulation of 9 genes of the TLR pathway and 5 genes from the chemokine (CC and the CXC) family. The chemokine, CCL8 was most upregulated by 60-fold. These studies used very high concentrations of Omniscan and free Gd, which is difficult to achieve in vivo.

**Key Points**

Gadolinium (both free and chelated form) can leak out into the tissues from injured vascular endothelium. Activation of incubated macrophages via MAPK/ERK and NF- *κ* B pathways was demonstrated in vitro using free Gd and very high concentrations of Omniscan.

## 5. Release of Proinflammatory Cytokines 

The fundamental principal underlying NSF is macrophage-stimulated release of inflammatory mediators. In vitro studies have shown significant elevations in proinflammatory cytokines, that is, interleukins such as IL-4, IL-6, IL-13, interferon-gamma (IFN-gamma), TGF-*β*, vascular endothelial growth factor (VEGF), and tumor necrosis factor-alpha (TNF-*α*) when incubated PBMC's are exposed to various concentrations of Gd-CAs (Figure 1) [43]. The in vivo study by Steger-Hartmann et al. [44] also supported the upregulation of cytokines in 51 randomized Hannover-Wistar rats exposed to various concentrations of gadodiamide (Omniscan titrated with gadolinium chloride). The rats were divided into 3 groups (A, B & C), and were exposed to daily incrementing doses of 2.5 mmol/kg of gadodiamide. They showed signs of NSF as early as after the third treatment and all of them had developed signs of NSF by day seven. Analysis of the cytokines revealed that thirteen of them were found to be elevated after the first administration. Most cytokines were chemoattractants for macrophages. Osteopontin, a potent inhibitor of vascular calcification, was the highest elevated cytokine by 6.8-fold. It also interacts with receptors CXCR4 and CCR7 of bone marrow derived fibrocytes, possibly activating them. However, it is unclear if this has any role in the pathophysiology of NSF. The other cytokines that were found to be elevated were IL-1*α*, MCP-1, tissue inhibitor of metalloproteinase (TIMP), and TGF-*β*. MCP-1 is considered a vasoactive factor which increases vascular permeability resulting in albumin leakage. An increase in TIMP is known to regulate the extracellular matrix and degradation. TGF-*β* and IL-1*α* presumably attract fibrocytes and promote fibrocyte differentiation. In addition, an increased release of proinflammatory cytokines, IL-*β*1 and MCP-1, was demonstrated in the bloodstream of subtotal nephrectomized (SNx) rats on repeated exposure to Gd-CAs [37]. Although the activation of macrophages has been studied exclusively in vitro, the release of pro-inflammatory cytokines has been demonstrated in in vivo experiments on rat models.

**Key Points**

Studies with incubated PBMC's, Hannover-Wistar rats and subtotal nephrectomized rats have confirmed the release of proinflammatory cytokines in response to exposure to Omniscan.

## 6. Cells Involved in Fibrosis

It is thought that proinflammatory cytokines and chemokines released from tissues can attract a circulating fibrocyte which expresses CD34 and procollagen-1 [45]. A circulating fibrocyte is derived from the bone marrow and is different from a mature “fibroblast” found in skin. These circulating fibrocytes have been shown to differentiate into myofibroblasts in fibrotic tissues. The cells involved in fibrosis are still under investigation. It has been postulated that resident fibroblasts, bone marrow-derived cells, endothelial progenitor cells, mesenchymal precursors and monocyte-derived fibrocytes may contribute to NSF [46].

**Key Points**

Cytokines when released attract circulating fibrocytes.

Resident fibroblasts, bone marrow derived cells, endothelial progenitor cells, mesenchymal precursors and monocyte-derived fibrocytes may contribute to NSF.

## 7. Fibrocyte Differentiation in Peripheral Blood 

Promotion of fibrocyte differentiation from peripheral blood monocyte in the presence of gadodiamide (Omniscan) has been demonstrated in vitro. The hypothesis is that gadodiamide suppresses the inhibitory signals (serum amyloid protein) for fibrocyte differentiation (Figure 1).

## 8. Fibroblast Activation

 Gd-CAs can stimulate collagen production from fibroblasts. Direct proliferative effects of gadodiamide (Omniscan) on in vitro NSF fibroblasts was validated by increased levels of collagen (1.8- to 4-fold) and hyaluronan (3.2- to 5-fold), change in fibroblast doubling time from 28 hrs to 22 hrs, and an increased expression of alpha-smooth muscle actin and vimentin [47]. Similar results were reproduced with lower concentrations of gadolinium chloride [48], and different classes of Gd-CAs. Edward et al. demonstrated that the concentrations required for linear gadolinium contrast agents, cyclic agents and reference gadolinium agents to produce maximum fibroblast proliferation were 0.1 mmol/L, 5 mmol/L and 0.01 mmol/L respectively [49]. The linear contrast agents (gadodiamide, gadoversetamide, gadopentate dimeglumine, and gadobenate dimeglumine) increased fibroblast cell number by 2.3-fold. Bhagavathula et al. [50] assessed two intracellular signalling pathways of an activated fibroblast, namely the MAPK and the phosphatidyl-inositol-3 kinase (PI-3K) pathways, by downstream production of phosphorylation of ERK and v-Akt murine thymoma viral oncogene (AKT) using human dermal fibroblasts (Figure 2(b)). The phosphorylated forms of ERK (i.e., p42 and p44) were increased up to 48 hours after exposure. However, an increase in phosphorylated AKT was not sustained after Omniscan exposure. Phosphorylated ERK also leads to an increase in matrix metalloproteinase-1 (MMP-1). In the presence of PI-3K and MAPK inhibitors, the deposition of type I procollagen and type I collagen was suppressed. Additionally in the presence of a PI-3K inhibitor, MMP-1 levels were inhibited while there was an unexpected upregulation of TIMP-1. The authors postulated that activation of ERK results in the transcription of MMP genes. PI-3K, by the production of AKT, plays a role on MMP modulation, perhaps involving the NF- *κ* B pathway. 

 Platelet-derived growth factor-tyrosine kinase (PDGF-TK) receptor is involved in myofibroblast proliferation and deposition of collagen. Interrupting earlier stages of PDGF-TK-receptor-mediated myofibroblast duplication is more advantageous than in the later stages when extracellular matrix has already accumulated in the fibrotic lesion. To assess the role of PDGF-TK receptor in the pathophysiology, Bhagavathula et al. [51] exposed six 2 mm full thickness punch biopsies from seven healthy subjects to concentrations of free gadolinium ranging from 0 mM to 20 mM and determined the levels of MMP-1, TIMP-1, type 1 procollagen and collagenolytic activity in these fibroblasts. When the cells were stimulated with PDGF, the responses were similar to gadolinium exposure with increases in TIMP-1 and MMP1 and little effect on type I procollagen. When a blocking antibody to PDGF-TK receptor was tested with gadolinium, the fibroblast proliferation had decreased from 47% to 37% indicating that PDGF-TK receptor may be involved in fibroblast proliferation. Inhibitors of PDGF-TK receptor may prove as effective interventions for fibrotic lesions.

**Key Points**

Gd-CAs and free Gd have proliferative effects on collagen in vitro fibroblasts.

The intracellular signaling pathways of an activated fibroblast are the MAPK and the phosphatidyl-inositol-3kinase (PI-3k).

PDGF-TK receptor may be involved in fibroblast proliferation on exposure to free gadolinium.

## 9. Role of TGF-*β* and Its Molecular Pathways in Fibrosis

 TGF-*β* is a profibrotic cytokine that attracts bone marrow derived fibrocytes, promotes their differentiation into myofibroblasts, increases synthesis of collagen, proteoglycans and prevents degradation of extracellular matrix by increasing TIMP-1 and plasminogen-activator inhibitor. It is secreted as native TGF-*β* and is proteolytically cleaved into a more active growth factor by transglutaminase. Parsons et al. [52] performed immunohistochemistry on five formalin-fixed, paraffin-embedded skin biopsies from patients with NSF. The cells stained for transglutaminases (TG-2) and factor XIIIa and showed strong reactivity to CD68 by spindle cells and histiocytes in the dermis and subcutaneous tissue in all the patients. The elevation in TG-2 and transglutaminase isopeptide confirms the cross-linking of glutamine and lysine residues of substrate proteins indicating transglutaminase activation. The authors speculated that TG-2-activated TGF-*β* increases gene expression of the extracellular matrix and decreases extracellular matrix proteolysis. Jiménez et al. [11] investigated the systemic nature of NSF by demonstrating elevated levels of TGF-*β* mRNA expression by in-situ hybridization in the skin, fascia, striated muscle and heart biopsies of nine NSF patients. TGF-*β* was expressed in CD68+/Factor XIIIa dendritic cells, and may be involved in the complex process of dendritic cell maturation. They also postulated that TGF-*β* may be involved in the multiplication and augmentation of more antigen-presenting cells. The TGF-*β* pathway has also been described by Kelly et al. [53]. Biopsy specimens from patients with NSF were evaluated via imunohistochemistry stains for TGF-*β*, angiotensin-converting enzyme (ACE), angiotensin II receptor-1(AT1), and p-Smad-2/3. Of the eleven biopsy specimens, 8 revealed the presence of TGF-*β* in the spindle cells, 8 stained for Smad-2/3, and 6 stained for both TGF-*β* and Smad-2/3. Faint staining of ACE was reported in 2 of the 11 specimens, while no AT1 staining was reported. All patients received at least 0.2 mmol/kg of Gd-CAs. A correlation was reported between dosage and expression of TGF-*β*. The authors postulated that the receptor AT1 was not involved, but other receptors in the renin-angiotensin system may play a role in NSF. A limitation of this study was that it evaluated AT1 and not AT2. It was suggested that matrix metalloproteinases, MMP-2 and MMP-9, thrombospondin-1, plasmin, and integrin alpha-v-*β*-6 activates TGF-*β* which then binds to a receptor complex leading to phosphorylation of Smad-2/3. These phosphorylated mediators then bind to Smad-4 and translocate to the nucleus. These actions can be inhibited by Smad-6 and Smad-7 (Figure 2(b)). Schieren et al. [54] reviewed the TGF-*β*-Smad-connective tissue growth factor (CTGF) axis in the skin biopsy specimens of 10 patients with NSF, 16 patients with systemic sclerosis, 8 patients without NSF on hemodialysis and 17 healthy controls. Patients without NSF on hemodialysis had a higher dermal expression of TGF-*β* mRNA and TIMP-1 in comparison to healthy controls, suggesting a profibrotic milieu in this group. The disproportionately increased levels of TGF-*β*, Smad2/3, CTGF, and TIMP-1 in patients with NSF suggest a profibrotic state, which may support fibroblast stimulation. Fretellier et al. [37] compared in vivo data on the biochemical effects of repeated administration of gadodiamide, non formulated gadodiamide, gadoterate, and saline to subtotal-nephrectomized (SNx) rats. Skin biopsies were taken at the end of the study (day 5) and to examine the long term effect of gadolinium, biopsies were also taken on days 11, 18 and 25. Immunohistochemical staining revealed CD34 and TGF-*β* distributed heterogeneously in the skin biopsies. The nonformulated gadodiamide group had worse skin lesions, microscopic evidence of inflammation, necrosis and extracellular matrix degradation, as well as CD34 and TGF-*β*_1_ stained cells. In contrast, the gadoterate group had no skin lesions or TGF-*β* staining.

**Key Points**

Skin biopsies from patients with NSF have elevated levels of TG-2 which activates TGF-*β*.

Increased TGF-*β* mRNA expression has been demonstrated in the skin, fascia, striated muscle and heart biopsies of patients with NSF.

Increased levels of TGF-*β*, Smad-2/3, CTGF and TIMP-1 in patients with NSF suggest a profibrotic state, which may support fibroblast stimulation in skin biopsies of patients with NSF.

Skin biopsies of SNx rats exposed to low stability Gd-CAs demonstrated CD34 and TGF-*β* staining cells.

## 10. Role of Metallothioneins

Metallothioneins (MTs) are cysteine rich proteins associated with cellular proliferation and programmed cell death. They are reservoirs for physiological metals such as zinc and copper and segregate potentially harmful metals such as cadmium, silver, mercury and arsenic. It is proposed that elevated levels of MT's in keratinocytes can lead to fibroblast proliferation (keloidogenesis) by inhibiting programmed cell death via zinc signaling cascades [55]. Metallothionein gene expression is modified in the secretome of fibroblast exposed to gadodiamide [56]. It is speculated that the gadolinium chelate may be directly inhibiting the metalloproteinase function as they have a zinc atom at the active site. Future research will help understand the role of metallothioniens in NSF.

**Key Points**

Metallothionein gene expression is modified in the secretome of fibroblast exposed to gadodiamide.

## 11. Role of Gd-CAs and Extracellular Matrix/Collagen Turnover 

It is well known that Gd-CAs may derange the enzyme/inhibitor system of collagen turnover. Collagen turnover is regulated by MMP-1 which breaks down extracellular matrix. MMP-1, also known as interstitial collagenase, has the capacity to cleave the triple helix of type I collagen allowing further collagen degradation. TIMP-1 antagonizes MMP-1. Alterations of collagen turnover are thought to be responsible for NSF development. An in vitro study [50] in which human dermal fibroblasts were exposed to two concentrations of Omniscan [50 mM and 250 mM] revealed elevated MMP-1 (2.7- to 3.3-fold), cell growth and TIMP-1 levels (1.9- to 2.3-fold) without any increase in type 1 procollagen. When these fibroblasts were exposed to TGF-*β*, there was an increase in type I procollagen (4-fold) and an increase in collagen (2-fold). MMP-1 was decreased by 50% in response to TGF-*β*. Type I collagen levels have been shown to be elevated in the cells after treatment with Omniscan. The levels of extracellular matrix regulators may be elevated in inflammation. Kelly et al. [57] proposed that TGF-*β* stimulates TIMP-1 which inhibits matrix metalloproteinases to break down the extracellular matrix causing fibrosis. They studied 16 formalin-fixed, paraffin-embedded specimens from 10 patients with NSF for the expression of TIMP-1, MMP-1 and alpha-smooth muscle actin. It was ascertained that TIMP-1 was strongly expressed in the cytoplasm of spindle cells in all the 16 samples. 13 samples had absent MMP-1. Alpha-smooth muscle actin was not seen in the spindle cells. In a study by Perone et al. [58], collagenolytic activity was suppressed in skin biopsies from normal subjects exposed to 50 mM of Omniscan. Punch biopsies from normal subjects were incubated and exposed to gadolinium. The supernatant was quantified for collagen fragmentation by sodium dodecyl sulfate-polyacrylamide gel electrophoresis (SDS-Page)-Coomassie brilliant blue staining and western blot technique by anti-MMP1-antibody to assess the levels of MMP-1. TIMP-1 and MMP-1-TIMP-1 complex levels were assessed by enzyme linked immunosorbent assay (ELISA). Piera-Velazquez et al. [59] demonstrated that Gd-CAs exposed NSF fibroblast showed increased production of collagen types I, III, fibronectin and hyaluronic acid which was sustained through different pathways. It was concluded that NSF fibroblast have a profibrotic phenotype and that collagen production by gadodiamide (Omniscan) was twice that of gadopentetate dimeglumine.

## 12. Role of Decorin and Fibrosis

Decorin is involved in collagen fibrillogenesis, growth factor modulation, fibrocyte differentiation and cellular growth which may perpetuate fibrosis. The role of decorin in the pathogenesis of NSF was studied by Gambichler et al. [60]. They assessed mRNA expression of decorin, versican and TGF-*β* in skin specimens of 10 patients with NSF, 16 patients with systemic sclerosis, 8 patients without NSF on hemodialysis and 17 healthy controls. They demonstrated that decorin was overexpressed in patients with NSF compared to patients with systemic sclerosis (*P* value < 0.014), hemodialysis patients (*P* value < 0.0074), and healthy controls (*P* value < 0.0001). It also correlated with TGF-*β* (*r* = −0.72) expression. Although decorin is antifibrotic, it is ineffective in patients with NSF as there is failure of upregulation of TNF-*α* or SMAD-7 expression.

## 13. Role of Fibroblast Growth Factor 23 and Klotho Protein 

High levels of serum phosphorus, 1, 25-dihydroxycholecalciferol (DHCC) and parathormone stimulate the release of fibroblast growth factor-23 (FGF-23) from osteocytes and osteoblasts. FGF-23, in turn, suppresses phosphate reabsorption in the kidney, inhibits 1-*α*-hydroxylase and parathormone secretion. The affinity of FGF-23 to its receptor complex is strengthened by an obligate coreceptor called klotho, a transmembrane protein produced by nephrons [61]. The extracellular domain of klotho is split by proteases to release “secreted klotho,” while in situ “membrane klotho” functions as the co-receptor to FGF-23. As CKD progresses, klotho's production decreases. Absence of klotho is associated with a phenotype of premature aging as shown in klotho knockout mice [62]. There is data associating normal aging with increasing levels of circulating fibrocytes [63]. Thus, CKD patients with a phenotype of premature aging may have an increased number of circulating fibrocytes. Conversely, secreted klotho, from klotho over expressing transgenic mice, can block type 2 TGF-*β* receptor expression in rat's renal epithelial cells and human embryonic kidney cells [64]. Patients with advanced CKD and reduced klotho may hence, loose the protective effective of blockade of the type 2-TGF-*β* receptor.

## 14. Permissive Factors Presumably Associated with the Development of NSF 

Although there is evidence in support of an association between gadolinium exposure and the development of NSF in end stage renal disease (ESRD) patients, there are a considerable number of in-vitro studies that further suggest that this association also involves the role of permissive factors. It is arguably intuitive to oversimplify that the development of this disease is as simple as an exposure to a gadolinium based contrast agent. If this was indeed the case, it is reasonable to conclude that far more patients would have developed NSF than there are reported cases. Although largely unproven in human subjects yet, one may hypothesize a scenario wherein patients are likely to develop NSF after exposure to gadolinium if the right environment exists. The following discussion is an attempt to summarize the permissive factors which were initially implicated in the development of NSF lesions. Some of them have been discounted in subsequent publications [65]. An extensive NSF-related literature search has surfaced notable permissive factors which can be broadly classified into associated proinflammatory conditions and an associated metabolic milieu as outlined in Table 1. 

## 15. Associated Proinflammatory Conditions

### 15.1. Role of Vascular Injury/Vascular Surgical Procedures

Earlier studies linked the possibility of vascular reconstructive surgical procedures to the onset of NSF in some individuals [15]. For example, of the 14 patients discussed by Cowper et al. in 2001, one of them who had chronic renal insufficiency was noted to develop NSF lesions following the repair of a leaking aortic aneurysm [1]. Other studies reported the development of NSF lesions following an acute occlusion of the dialysis access [14, 66], creation of an arteriovenous fistula [67, 68] and post angioplasty [67]. The onset of symptoms was noted to be closely related to either an organ transplantation or placement of central catheters in approximately 90% of the NSF patients [69]. Macrovascular injuries such as thromboembolic events and vascular surgery, and microvascular injuries such as malignant hypertension and vascular rejection were reported prior to the development of NSF lesions [70]. In order for this theory to be true, there must be interactions between the vascular endothelium and fibrocytes that contribute to the fibrosing reaction seen with NSF. Vascular injury can cause platelet activation which can then interact with one another and attach themselves to the exposed vascular endothelium. This in turn, may predispose a patient to develop NSF by stimulating the production of profibrotic factors that further enhance collagen synthesis by attracting fibrocytes and by releasing CD34^+^ progenitor cells [46]. Gd-CAs exposure in a patient with ESRD and endothelial dysfunction or damage may act as an additional trigger to initiate the process of NSF [71]. This can occur by one of two possible mechanisms. First, a damaged vascular endothelium may facilitate dissociated Gd^3+^ ions to enter the interstitial space and tissues. Second, the associated inflammation can stimulate the production of profibrotic cytokines which can then lead to the attraction of circulating fibrocytes to tissues containing Gd^3+^ ions, causing an increase in the collagen production leading to fibrosis [72]. 

### 15.2. Role of Thrombotic Events/Procoagulant States

The presence of right atrial clots, thrombosed veins from indwelling catheters, thrombosed vascular accesses, pulmonary embolism [73], renal vein thrombosis of the transplanted kidney [22], peripheral vascular occlusion, transient ischemic attacks, multiple brain infarcts [66], an episode of atheroembolism [2], deep vein thrombosis, vena caval thrombosis, procoagulant states such as antiphospholipid antibody syndrome [2, 74], and protein C deficiency [5] were noted in high frequency in patients with NSF. Hypercoagulability and thrombotic episodes were also associated with NSF in 12% of patients [14]. Based on these observations, the role of a thrombotic event and procoagulant state can be regarded a two-hit hypothesis; vascular and endothelial dysfunction is a primer waiting for a second trigger to set forth the fibrosing event [75]. 

### 15.3. Severe Infection

Although a limited study, some investigators have hypothesized that an infection may be a predisposing factor for the development of NSF after exposure to gadolinium-containing contrast agents [76]. In an observational study of eight patients, the association of NSF with infection status, both focal and systemic, was found to be highly significant (*P* < 0.0001). They found a high rate of infection at the time of magnetic resonance imaging contrast administration in a small group of patients who later developed NSF. Furthermore, for patients without infection, the association of NSF with renal failure was also noted to be highly significant (*P* < 0.0001). It is believed that the presence of an infective state triggers cytokine proliferation which, in turn, stimulates circulating fibrocytes leading to NSF. However, Lauenstein et al. noted in their retrospective study that the 2 out of the 14 NSF patients with associated acute peritonitis showed no significant relationship between severe infection and NSF [77].

### 15.4. Chronic Hepatitis C, Hepatic Disease and Liver Transplantation

A few case reports have suggested a link between NSF patients and an associated history of hepatic disease or chronic hepatitis C infection followed by liver transplantation [78]. This association did not appear to have been upheld in subsequent reports since. Mazhar et al. [79] noted that greater than 97% of NSF patients had associated severe renal insufficiency irrespective of the presence or absence of concomitant liver disease. It was also concluded that the presence of liver disease or liver transplantation did not increase the risk of developing NSF. Another recent study has shown that only 0.1% (1/709) of liver transplantation patients exposed to gadolinium based contrast agents or 1.4% (1/74) of CKD stage 5 patients requiring dialysis had biopsy proven NSF. This is comparable to the reported incidence of NSF in ESRD patients regardless of liver transplantation [80]. Therefore, liver transplantation may not necessarily be an additional risk factor in the development of NSF.

### 15.5. Hyperparathyroidism

Gadolinium can actively compete with calcium for cation binding sites on cellular membranes and can bind to and activate the extracellular calcium-sensing receptor, present on multiple cells and tissues such as fibroblasts, kidney, parathyroid glands, hepatocytes, and pancreas [71]. In an autopsy case series study, two NSF patients had enlarged parathyroid glands and two other patients were reported to have high serum parathyroid hormone levels [81]. However, this association does not hold in healthy volunteers since studies have failed to show a definite relationship between serum calcium levels and exposure to Gd-CAs. Since in vitro studies have demonstrated an increase in the release of bone marrow derived cells such as CD34^+^ progenitor cells into peripheral circulation following administration of parathyroid hormone [82, 83], it is possible that hyperparathyroidism may play a role in the pathogenesis of NSF. More studies are needed to confirm this possible association. 

### 15.6. Hypothyroidism

There has been speculation about the link between hypothyroidism and the development of NSF since hypothyroidism can increase the diffusion of gadolinium into the interstitial spaces, recruit circulating fibrocytes, and result in an increase to the exposure to free gadolinium. A significant association, however, could not be established in a multivariable analysis [84]. Interestingly, a recent case report discussed the selective malabsorption of oral levothyroxine in a hypothyroid patient who presumably developed NSF of the gastrointestinal tract [85].

## 16. Associated Biochemical Milieu

### 16.1. Acidosis

The relationship between Gd-CAs exposure, acidosis and the development of NSF is unclear. Acidosis may, in theory, increase the susceptibility of ESRD patients to NSF since its presence is likely to promote transmetallation with the liberation of free Gd^3+^ ion from the chelate. In a study by Grobner, five NSF patients had a mean pH value of 7.29 ± 0.04, and a mean bicarbonate value of 19.5 ± 1.7 mmol/L suggesting the role of metabolic acidosis in the pathogenesis of NSF. They advocated correction of metabolic acidosis prior to administration of gadolinium-based contrast agents in patients with end-stage renal disease [23]. The phosphate binder, sevelamer hydrochloride, although not significantly absorbed systemically, has been shown to cause metabolic acidosis in ESRD patients. Sevelamer, when binding phosphate in the gut, releases hydrochloric acid and induces metabolic acidosis in a dose-dependent manner. Concurrent sevelamer use has been documented in multiple cases of NSF [5]. On the contrary, Khurana et al. reported normal serum bicarbonate levels in 6 cases of NSF associated with gadolinium based contrast agents. However 5 of these 6 patients had an increased anion gap [67]. Furthermore, there are several documented cases of NSF in individuals with normal or near normal bicarbonate levels [86, 87]. Based on these contradicting reports, it still remains unclear if metabolic acidosis influences the development of NSF. In the case of linear chelates, the rates of dissociation were noted to obey an acid-catalyzed behavior involving monoprotonation and diprotonation of the gadolinium chelate followed by dissociation of the Gd^3+^ ion. The rates of dissociation of cyclic ligands are much slower than their linear counterparts. Although a theoretical possibility, the degree of acidosis is too small to have any significant impact on the kinetic rate of dechelation [88]. Moreover, other studies did not find a significant relationship between acidosis and NSF [86, 87]. A definite link between acidosis and NSF remains to be established.

### 16.2. Iron

Intravenous iron, a frequently incorporated therapy in anemia protocols for ESRD patients, may interact with Gd-CAs administration and hence be considered a permissive factor for the development of NSF. There are two theories which may explain the role of iron in the development of NSF. First, there may be an exchange of ligand (L) between Gd^3+^ and Fe^3+^ ions as follows:

(1) $$\begin{array}{c} \left(\text{GdL}+{\text{Fe}}^{3+}\Leftrightarrow {\text{Gd}}^{3+}+\text{FeL}\right) \end{array}$$

Gd^3+^ may precipitate in the tissues in the form of gadolinium phosphate that could be phagocytosed by macrophages. In order to explain the increased iron levels in such patients, Swaminathan et al. [89] suggested that gadodiamide exposure in patients with renal insufficiency may result in a substantial decrease in total iron-binding capacity and increased iron mobilization, resulting in transferrin oversaturation. Iron mobilization may in theory, lead to transmetallation and release of Gd^3+^. Similarly, Panesar et al. [90] demonstrated that there was no statistically significant difference in the serum iron, total iron-binding capacity, ferritin, or transferrin saturation before and after exposure to Gd in six out of 25 dialysis patients who had a magnetic resonance imaging (MRI) scan. In an average follow-up period of 20 months, none of these patients developed NSF despite a history of exposure to Gd. They inferred that in order for iron mobilization to occur, the presence of at least another confounding factor or sufficient iron store is required in the presence of Gd. Second, free gadolinium and catalytic iron may synergistically coordinate, resulting in oxidative stress, inflammation and tissue injury. Serum iron levels were noticed to increase in the inflammatory syndrome. Hope et al. [91] demonstrated that rats when treated with high dose gadodiamide (Omniscan, GE Healthcare), erythropoietin (EPO) and intravenous iron developed severe fibrotic lesions. It is likely that tissue levels similar to those seen in iron overload states were achieved, resulting in increased oxidative tissue damage and increased deposition of gadolinium. As a further evidence to support the role of iron, large amounts of gadolinium were detected on paraffin embedded tissue specimens from NSF patients using energy dispersive spectroscopy. Along with Gd, large deposits of iron were also identified in the same tissue samples [92]. Miyamoto et al. used Berlin blue staining on the skin biopsies from nine japanese patients who had NSF and found that six of them had specific deposition of iron in the fibrocytes or between the collagen bundles around blood vessels [93].

### 16.3. Erythropoietin

EPO therapy can promote the pathogenesis of NSF in multiple ways. EPO has the ability to induce endothelial dysfunction by inhibiting dimethylarginine dimethylaminohydrolase, thereby increasing asymmetric dimethyl arginine [46]. It also has stimulatory effects on the vascular endothelium, smooth muscle cells and platelets causing endothelial and progenitor cell proliferation. It can also induce inflammation directly since administration of EPO has been shown to release vasoactive factors such as monocyte chemoattractant protein-1, endothelin-1, thromboxane A2 and selectin. In vivo studies have demonstrated EPO causing accelerated fibrin-induced wound healing [94]. EPO can lead to the recruitment of bone marrow derived circulating fibrocytes and also potentiate fibrocyte activity through EPO receptors on fibrocytes [91]. An in vivo experiment was conducted by Hope et al. to determine the effect of gadodiamide (Omniscan, GE Healthcare), intravenous iron and EPO on Hannover-Wistar rats (Crl: WI [Han]). Rats treated with gadodiamide and both EPO and intravenous iron had significantly worse skin lesions at gross and histologic analysis (*P* = 0.004) when compared with the rats treated with gadodiamide only. They also had increased levels of deposited gadolinium (*P* = 0.012) [91]. When given with gadodiamide, EPO may decrease inflammation through iron metabolism pathways by removing poorly ligated iron and limiting oxidative stress but when given in the setting of increased iron stores, it may, quite to the contrary, promote inflammation. In many documented cases of NSF, patients were also receiving erythropoietin [5, 95, 96]. EPO resistance could develop in a prolonged inflammatory state as seen in NSF [97]. Swaminathan et al. suggested that this could be the reason for the high dose erythropoietin requirement in these patients. Patients with nephrogenic fibrosing dermopathy were noted to have received a significantly higher dose of erythropoietin than control patients (427 U/kg of body weight per week [range, 66 to 1195 U/kg per week] versus 198 U/kg per week [range, 14 to 720 U/kg per week]; *P* < 0.001) [98]. The mechanistic theory underlying this remains in the observation that EPO can stimulate hematopoietic progenitor cell translocation to the circulation to induce a fibrogenic response. Therefore, high-dose EPO may contribute independently to the development of NSF [99]. With the implementation of the Dialysis Quality Outcome Initiative for anemia [91] in 1997, high-dose EPO became widespread which, coincidentally, is the same year the first cases of NSF were also noted. Given the proinflammatory nature of erythropoietin (cause) and the use of high-doses of EPO in NSF which by itself is an inflammatory state (effect), the precise pathophysiological role of this permissive factor still remains largely unknown. 

### 16.4. Calcium and Phosphate

When competing for the gadolinium chelate, calcium ions behave similar to ferric ions in that displacement of free Gd^3+^ occurs via transmetallation and perhaps even more readily since the ion radius of Gd^3+^ is closer to that of calcium. In vivo experiments have shown rapid precipitation of free Gd following intravenous injection of gadolinium chloride where it is phagocytosed by macrophages [88]. On the contrary, a study by Khurana et al. observed an inverse relationship between serum calcium and tissue gadolinium levels [100]. 

The role of hyperphosphatemia in the pathogenesis of NSF could be explained by two theories. First, the release of Gd^3+^ from non-ionic linear Gd-CAs was noted to be 10 times higher than from their ionic linear counterparts due to the differences in their thermodynamic stability constants. Frenzel et al. [101] have demonstrated a 75% increase in the release of free Gd ions from gadodiamide (Omniscan) and gadoversetamide (Optimark) on the addition of phosphorus. On the other hand, cyclic Gd-CAs remained stable at both normal and elevated phosphate levels. In support of this hypothesis, Fretellier et al. [102] have demonstrated the profibrotic effects of gadodiamide, in the presence of hyperphosphatemia in renally impaired rats. Second, it is also proposed that free Gd^3+^ in the presence of hyperphosphatemia due to renal dysfunction precipitates as gadolinium phosphate in the tissues. Experimentally, it has been shown that high doses of lanthanides (including Gd) exceed the complexing ability of plasma and form insoluble precipitates with hydroxides, phosphates and carbonates. Abraham et al. [103] noticed high Gd or calcium in tissue deposits of NSF with increasing gadodiamide dose indicating that Gd was released from its chelate as free Gd^3+^ ion. To add to the controversy, it is currently unknown whether hyperphosphatemia or the phosphate binders such as sevelamer or lanthanum pose a greater risk. No specific recommendations regarding the continuation or discontinuation of these chemical agents have been made so far [104]. Increased levels of ionized calcium hence, in theory, would increase the risk of transmetallation and increased phosphate levels not only would enhance this process but also precipitate free Gd. Retention of gadolinium in the body would consequentially lead to the process of fibrosis in NSF patients [105]. Interestingly, persistent hypocalcemia was deemed to have reduced the chances of transmetallation of Gd^3+^ ions and development of NSF lesions in an ESRD patient with demonstrable insoluble gadolinium in the dermis [106].

In summary, many pathophysiological mechanisms have been proposed for NSF. Transmetallation, release of cytokines by the activation of skin macrophages, production of collagen, extracellular matrix and myofibroblasts by fibrocytes have all been demonstrated conclusively in animal experiments. There are intriguing theories of the role of decorin, metallothionein, osteopontin and gadolinium-stimulating TG-2 leading to the production of active TGF-*β*. A novel theory is the process through which klotho reduction in CKD patients induces a premature aging phenotype and causes a profibrotic state. PDGF-receptor-mediated activation of fibroblast is a promising theory as PDGF receptor inhibitors may be used to target NSF lesions. Some of the aforementioned permissive factors have been implicated in in vitro studies. However, further studies in humans, which would take into account their role as a combined entity, are deemed necessary to provide a definite explanation to this rather interesting, yet complicated disease. 

## Figures and Tables

**Table 1:** Permissive factors presumably contributing to the development of NSF lesions.

Associated proinflammatory conditions	Associated biochemical milieu
Vascular injury/vascular surgery procedures	Acidosis
Thrombotic events/procoagulant states	Iron
Severe infection	Erythropoietin
Chronic hepatitis C, hepatic disease and liver transplantation	Calcium and phosphate
Hyperparathyroidism	
Hypothyroidism	
